# Adsorption of Methylene Blue on Activated Carbon Surfaces Obtained by Shock Compression of Graphite Using Reactive Molecular Dynamics

**DOI:** 10.3390/molecules29246030

**Published:** 2024-12-21

**Authors:** Tomasz Panczyk, Pawel Wolski, Krzysztof Nieszporek, Robert Pietrzak

**Affiliations:** 1 Jerzy Haber Institute of Catalysis and Surface Chemistry, Polish Academy of Sciences, ul. Niezapominajek 8, 30239 Cracow, Poland; pawel.wolski@ikifp.edu.pl; 2 Department of Theoretical Chemistry, Institute of Chemical Sciences, Faculty of Chemistry, Maria Curie-Sklodowska University, Lublin pl. Maria Curie-Sklodowska 3, 20031 Lublin, Poland; krzysztof.nieszporek@umcs.pl; 3 Department of Applied Chemistry, Faculty of Chemistry, Adam Mickiewicz University in Poznań, Uniwersytetu Poznańskiego 8, 61614 Poznań, Poland; pietrob@amu.edu.pl

**Keywords:** shock compression, activated carbon, methylene blue, molecular dynamics

## Abstract

This study explores the formation of functionalized carbon surfaces through shock compression of graphite in the presence of water, modeled using molecular dynamics and the ReaxFF reactive force field. The shock compression method produces activated carbon with surface functionalities, primarily hydroxyl groups, and varying morphological properties. Two approaches, unidirectional and isotropic compression, yield distinct surface structures: the former preserves a relatively flat surface, while the latter generates corrugated features with valleys and ridges. These features significantly impact the adsorption properties of methylene blue (MB), a commonly used dye. Simulations reveal that MB molecules are highly mobile on flat surfaces, aligning with a mobile adsorption model. However, on corrugated surfaces, MB exhibits localized adsorption, with the deepest valleys effectively immobilizing the dye molecules. Additionally, the study highlights the influence of surface hydroxyl groups, which, through interactions with water molecules, prevent MB from occupying these regions. The findings underscore that traditional adsorption models may not fully capture the dynamics of MB adsorption on activated carbons with complex morphologies. These insights are critical for advancing carbon-based adsorbents in water purification applications.

## 1. Introduction

The removal of dyes from water is crucial, due to their harmful environmental and health impacts. Dyes can be toxic, carcinogenic, and non-biodegradable, making them persistent pollutants in water systems. Adsorption methods offer an effective, low-cost, and versatile solution for dye removal, enabling the efficient capture of dye molecules from wastewater. Using adsorbents such as activated carbon ensures that contaminants are retained on the adsorbent surface, preventing their release back into the environment [1,2,3].

Though adsorption is widely used for water treatment due to its simplicity, efficiency, and safety, it does not alter pollutants chemically, which can limit its effectiveness. Photocatalysis transforms harmful pollutants into less toxic substances, offering advantages like low energy consumption and no secondary pollution, though it requires specific conditions and materials. Microwave catalysis is an emerging technique that enhances reaction rates through selective heating, but its application can be constrained by the high cost of equipment and energy [4]. Therefore, adsorption remains one of the most effective methods for eliminating organic pollutants. The adsorptive removal of antibiotics such as tetracyclines from water is a highly effective approach to mitigating their environmental impact, as advanced adsorbents like MIL-88B(Fe) can significantly enhance both pollutant enrichment and catalytic degradation capabilities [5].

Methylene blue (MB) is commonly used in the textile and chemical industries, and its discharge into water bodies can harm aquatic ecosystems and pose health risks to humans. As a persistent organic dye, MB is resistant to natural degradation, making it challenging to remove through conventional treatment processes. Effective removal methods, such as adsorption, are critical for preventing contamination and safeguarding water quality [2,6]. Activated carbons are excellent materials for removing MB from water due to their high surface area and strong adsorption capacity. Their porous structure allows for the efficient trapping of dye molecules, making them highly effective for water purification applications [7,8]. Therefore, many papers have been published annually describing the adsorption isotherms and kinetics of various dyes on many types of activated carbons obtained from various sources. Modeling studies, especially molecular dynamics analyses, are substantially less popular, but we can still find several interesting contributions, though molecular models of activated carbon are usually limited to slit-like pores composed of functionalized graphene sheets [9,10,11,12].

The advanced modeling of activated carbon structures is challenging due to the variety of possible morphologies of carbon atom arrangements, but knowledge of this kind is important for a proper understanding of adsorption phenomena occurring in these materials. The available approaches can be divided into constitutive models and reconstruction and mimetic methods of constructing 3D carbon structures from single atoms [13]. The constitutive methods rely on the combination of some pre-defined building blocks such as platelets [14], small domains of stacked graphene nanocrystals [15], or fullerene fragments [16,17] finally giving a structure with the required porosity or other properties of interest. The reconstruction methods use (hybrid) reverse Monte Carlo simulations [18,19] to reconstruct some experimentally available property of the material (pore size distribution function, for instance) [20]. Mimetic methods rely on replicating the actual synthesis processes at the atomic level. These simulations involve heating and quenching carbon atoms, allowing them to form disordered structures that mimic real-world materials. This approach provides valuable insights into the structure–property relationships of disordered carbons and aids in the design of materials with tailored properties [13]. The mimetic approach requires reactive force fields like ReaxFF [21] or AIREBO [22], for instance, to produce bonds between atoms during the mimetic procedure.

In the current study, we will apply an approach to constructing disordered activated carbon structures that, to the best of our knowledge, has not been explored in this context. This involves shock compression of graphite in contact with water to produce oxygen-containing surface functional groups when combined with a reactive force field. Shock compression [23] mimics a shock wave passing through the material and can be efficiently modeled using the Hugoniot equation of state, as described in more detail in the Section 3. Thus, this approach belongs to the mimetic methods, although the application of shock compression is not a common method for producing activated or disordered carbons. However, as we will show soon, the resulting structures are representative of samples obtained using other methods, like reverse Monte Carlo, for instance.

Shock compression has been effectively employed to model degradation reactions in various plastic materials, such as low-density polyethylene [24], polypropylene, and polystyrene [25]. This method enables controlled transformations in the chemical state of the material by adjusting the compression pressure. Consequently, the application of this technique to the construction of disordered carbon structures offers a promising alternative to quenching or reverse Monte Carlo methods, as discussed in the following sections. Shock compression has been widely used, often in conjunction with reactive molecular dynamics force fields such as ReaxFF, to study the mechanical properties of various materials. Computational studies have specifically investigated the response of semiflexible polymers to the shock front direction [26]. Moreover, it has been demonstrated that the principal shock Hugoniot method shows good agreement with experimental data up to 50 GPa for two hydrocarbon polymers, polyethylene (PE) and poly(4-methyl-1-pentene) (PMP), when using ReaxFF [27].

The article is organized as follows: We first examine how functionalized activated carbon surfaces can be generated from ideal graphite through shock compression in the presence of water. Next, we utilize these generated surfaces to model the adsorption of MB from aqueous solutions. We demonstrate that, depending on the application method of the shock wave front, the resulting surfaces can be nearly flat (with hydroxyl-containing groups) or highly irregular. The adsorption of MB on these surfaces will follow either the mobile or localized adsorption model. Additionally, we show that areas with a high density of hydroxyl groups become inaccessible to MB. Finally, we discuss the binding energy of MB at various adsorption sites on the functionalized activated carbon surfaces produced by shock compression.

## 2. Results

### 2.1. Formation of Functionalized Carbon Surfaces Using Shock Compression and ReaxFF Force Field

The starting point of this study is the generation of functionalized carbon surfaces from an ideal graphite slab structure. For this purpose, we subjected a 6-layered graphite slab with initial dimensions of 5.883 nm × 6.794 nm to shock compression using the Hugoniostat dynamics method [23], targeting a compression pressure of 40 GPa. The slab was periodic in the x and y directions and was in contact with liquid water in the z direction. The choice of this compression pressure range was dictated by the requirement that the reactions between water molecules and carbon atoms should start and proceed in a controlled manner. Lower compression pressures did not initiate reactions, while significantly higher pressures could lead to massive reactions and eventually cause the computations to crash.

**Figure 1:**
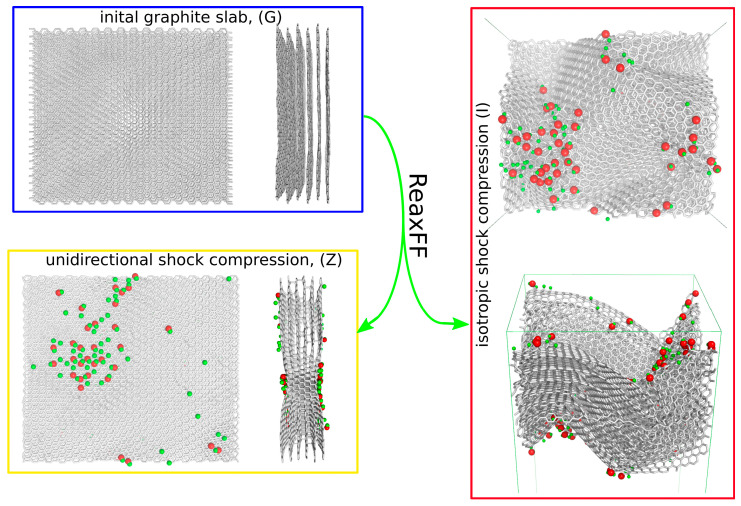
Graphical representations of the initial graphite slab (blue frame) used in subsequent computations with the ReaxFF force field shown, with shock compression applied both in the direction normal to the graphite surface (yellow frame) and isotropically in all three Cartesian directions (red frame). The applied compression pressure was 40 GPa. Carbon atoms are depicted as gray sticks, oxygen atoms as red spheres, and hydrogen atoms as green spheres.

The application of the shock wave to the simulation box can be conducted isotropically, meaning compression is performed in all x, y, and z directions, in a single direction or in two coupled directions. In the case of the graphite slab, the most interesting scenario would be the application of a shock wave perpendicular to the graphene plane or isotropically. Figure 1 graphically outlines the essential results for both methods of shock wave application.

Reactions with oxygen and hydrogen, originating from water, as well as internal transformations of the graphite structure, can occur due to the application of the ReaxFF reactive force field for all elements in the simulation box. Because the system is subjected to very high pressures and temperatures, the applied parameter set of ReaxFF was representative of the combustion branch of CHO (carbon–hydrogen–oxygen) systems [28]. The full parameters set is also attached in the supplementary information file.

**Figure 2:**
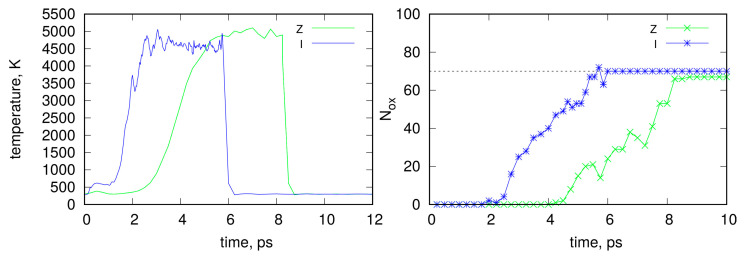
The **left graph** shows the temperature evolution during shock application and subsequent relaxation in the graphite slab for the unidirectional (Z) and isotropic (I) cases. The **right graph** depicts the corresponding number of oxygen-containing groups, *N_ox_*, on the surface during these computation stages.

The initial structure of the graphite slab is shown in Figure 1. For clarity, the water molecules are not explicitly displayed; however, in the computations, 2464 water molecules surrounded the slab above and below, while the sides of the slab were periodically replicated using periodic boundary conditions. The application of shock compression led to a rapid increase in pressure (stress components) and a gradual increase in temperature, as shown in Figure 2. This resulted in either the deformation of the graphite slab or the formation of new chemical bonds between carbon atoms, as well as the functionalization of carbon atoms by oxygen and hydrogen from water molecules.

As shown in Figure 1, the direction or style of shock wave application resulted in significantly different morphologies of the shocked samples. The degree of chemical or mechanical alteration of the sample also depends on the duration of shock wave application. Initially, the number of new chemical connections and mechanical deformations is small, but they gradually increase until the sample undergoes complete chemical and mechanical reconstruction. Figure 2 shows the time dependence of the system temperature during shock application and the subsequent relaxation after reaching the target state. As observed, the temperature rapidly increases to approximately 5000 K within several picoseconds, and the method of shock application influences the rate of this temperature rise. The formation of new oxygen-containing groups on the surface correlates with the temperature increase.

For this study, we selected timeframes that revealed a similar and relatively small number of surface hydroxyl groups, *N_ox_*, approximately 70 on both sides of the slabs. Thus, after reaching approximately 70 *N_ox_*, the shock was stopped, and the system was allowed to relax. This choice of functionalization level allowed us to study surfaces with distinct, highly functionalized areas and neighboring areas, without chemical functionalization but with mechanical deformations, as depicted in Figure 1. This approach highlights an intriguing behavior of MB adsorption on such mixed hydrophobic/hydrophilic surfaces, as discussed in the subsequent sections.

The unidirectional shock led to the preservation of the slab’s nearly unaltered horizontal shape, but resulted in the local development of crosslinks between successive graphene layers. This was accompanied by the rehybridization of carbon atoms and the formation of a local diamond-like structure within the slab, along with the simultaneous formation of a shallow depression. Interestingly, the area where the crosslinks formed was also extensively functionalized by surface hydroxyl groups (red and green spheres). Other areas were almost free of functional hydroxyl groups or revealed a small amount of hydrogen terminal atoms. A closer visual inspection of these extensively functionalized areas, as shown in supplementary information Figure S1, leads to the conclusion that the functional groups on the surface are exclusively hydroxyl-type, with oxygen atoms linked to rehybridized sp2 to sp3 surface carbon atoms, and a small number of hydrogen atoms linked directly to surface carbon atoms. These functionalized areas of the graphite surface will exhibit different properties since oxygen and hydrogen atoms carry partial charges of approximately −0.6e and +0.3e, respectively, while typical (distant from oxygen) carbon atoms have essentially no charge.

Recent density functional theory (DFT) calculations [29] on the composition of oxygen functional groups on graphite surfaces revealed that at room temperature and within a pH range of 0 to 14, the edge surfaces of graphite are fully oxygenated, while the basal sites remain unsaturated. The oxygen functional groups at the edge sites predominantly consist of hydroxyl and ketonic groups, with carboxyl and carbonyl groups present in smaller amounts. Furthermore, surface Pourbaix diagrams indicated that the oxygen functional groups at the edge sites are primarily composed of hydroxyls and ketones. In our study, we did not observe ketone groups. This is likely due to the fact that the reactions were restricted to the basal graphite planes, unlike the edge sites. However, the most probable explanation is the specific conditions applied in our study—extreme pressure and temperature combined with an excess of water. Under such conditions, the dehydrogenation of surface hydroxyls, which would lead to the formation of ketones, is unlikely.

Isotropic shock compression leads to substantial changes in the bulk structure of graphite, as well as chemical functionalization of the surface. Interestingly, major functionalization occurred at the ridges or slopes, but not in the valleys. Additionally, the variety of functional groups increased, with carbonyl and ether/epoxy groups appearing alongside hydroxyl groups, as shown in Figure S2. However, their number is relatively small compared to the dominant amount of hydroxyl groups. Thus, from a chemical perspective, the predominant reactions are substitution reactions, where double bonds break and form links with hydroxyl groups from water molecules. The distribution of oxygen-containing functional groups will lead to different surface properties between the ridges and valleys, due to the charges carried by these oxygenated species. Consequently, it is expected that such a surface will exhibit heterogeneity in terms of the adsorption energy of other molecules.

The two approaches to shock compression application led to different surface morphologies, as discussed. The unidirectional approach resulted in a structure similar to the so-called Sterling FT carbon black model, a graphitized activated carbon model. The atomic structure of Sterling FT carbon has been recently constructed using hybrid reverse Monte Carlo simulations [18], starting from graphite and using wide-angle X-ray scattering data as reference structural data and applying a linear temperature increase up to 5000 K [20]. However, Sterling FT does not exhibit diamond-like intrusions or oxygen functionalities. Our approach, using shock compression with the ReaxFF force field, can be easily tuned to produce fewer complex states by reducing compression pressure and modifying the environment. In contrast to models of functionalized carbons based on the assumption of graphitized slit-like pores with manually added functional groups [30,31], our approach leads to functionalization as a result of the initiation of chemical reactions due to the energy input caused by compression and temperature.

The structure obtained through isotropic compression is similar to the structural model of morphologically disordered slit-shaped carbon pores. This model was recently reconstructed [20] using LMA10 as a reference material, along with temperature-quenched Monte Carlo simulations that employed a three-body environment-dependent interaction potential (EDIP), parameterized for carbon–carbon interactions [32]. In another study [19], a similar morphology of activated carbon was achieved using hybrid reverse Monte Carlo simulations, the EDIP [32], and Erhard–Albe force fields [33]. Our approach, which allows for tuning both the morphological disorder of the sample and its chemical functionalization, enables us to easily produce structures with less disorder, as in ref [20], or with even more disorder, as in ref [19].

**Figure 3:**
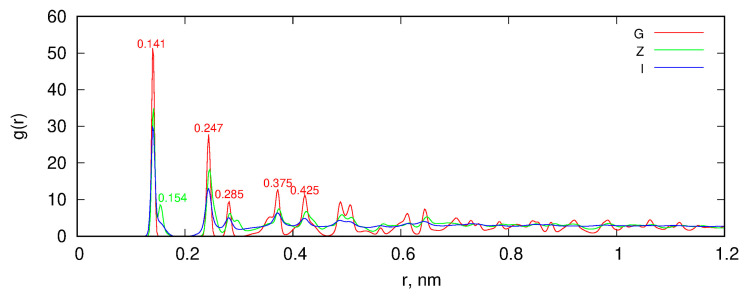
Radial distribution functions (RDF) between carbon atoms for the samples shown in Figure 1. G represents the intact graphite slab, Z denotes the graphite slab after unidirectional shock compression along the z direction, and I corresponds to the graphite slab after isotropic shock compression.

Figure 3 shows the radial distribution functions (RDF) between carbon atoms for each sample depicted in Figure 1. The successive curves illustrate how the graphite structure progressively changes after shock compression. The red labels correspond to distances characteristic of graphite; the first three peaks represent atomic distances within an aromatic ring, while the next two indicate interlayer distances in graphite. However, these distances should be interpreted as distances between atoms rather than between layers.

Unidirectional shock compression preserves the positions of the peaks marked in red, although these peaks become more diffuse compared to those for intact graphite. The green label indicates distances associated with a diamond-like structure, which emerge in samples after unidirectional compression. The RDF for isotropic compression reveals more diffuse peaks corresponding to graphite, and beyond 0.5 nm, any periodicity in the positions of carbon atoms is lost. This RDF closely resembles the RDF obtained from quenched molecular dynamics simulations or models reconstructed using hybrid reverse Monte Carlo methods, as reported by Palmer and Gubbins [13].

### 2.2. Adsorption of Methylene Blue

**Figure 4:**
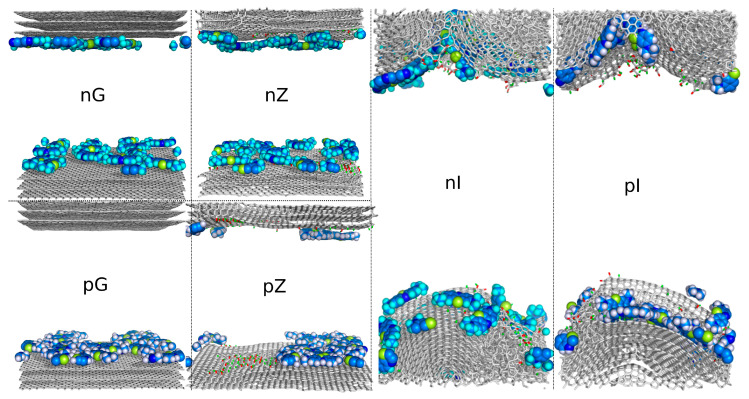
Simulation snapshots of MB adsorption in slit-like pores formed by either intact graphite slabs or after shock compression. The labels on each graph indicate which form of MB was used (n for MBn, p for MBp), while the capital letters G, Z, and I represent the type of solid substrate: G for intact graphite, Z for unidirectionally compressed graphite, and I for isotropically compressed graphite. The snapshots depict the simulation boxes after 80 ns simulations, with 20 MB molecules inserted in bulk water at T = 300 K and p = 1 bar. The color codes are as follows: gray sticks for surface carbons, red sticks for surface oxygen, green sticks for surface hydrogen, blue spheres for MB carbons, deep blue spheres for MB nitrogens, yellow spheres for MB sulfur, cyan spheres for MBn hydrogens, and white spheres for MBp hydrogens.

The obtained surfaces were used to construct slit-like mesopores for analyzing MB adsorption at various pH levels, i.e., below and above pKa = 3.8 [6]. Figure 4 shows simulation snapshots of the final simulation frame for each studied system. The graphs in Figure 4 are labeled using one lowercase and one uppercase letter. The lowercase letter indicates which form of the dye was used, while the uppercase letter defines the type of surface, as explained in the caption of Figure 4.

Analysis of the MD trajectories led to several qualitative conclusions. First, all dye molecules, initially placed in bulk water at a distance beyond the interaction cutoff from the surface, were caught and adsorbed onto the surfaces. They were distributed randomly between the upper and lower surfaces, and in the case of pG, all MBp molecules ended up on the lower surface. This does not imply that the surfaces are not equivalent—it is simply a result of random configuration. Another conclusion is that adsorption in all studied cases is strong, as no spontaneous detachment of dye molecules from the surface was observed after adsorption. Additionally, the corrugated surface (I—after isotropic compression) primarily attracted the dye molecules to the valleys, while flat surfaces exhibited high mobility of adsorbed molecules. Finally, the dye molecules appeared to avoid highly functionalized areas of the surfaces. These conclusions are further verified through more precise quantitative analyses.

Apart from surface–dye interactions, the behavior of dye molecules in water (without a surface) was analyzed in separate simulations. The key results are shown in the supplementary information file in Figure S3. The oxidized form of MB, present at a neutral pH and above, denoted as MBn, exhibits a tendency to form ribbon-like structures. This behavior is typical for bisazo dyes, such as Congo Red or Evans Blue [34]. The size of these ribbons is limited to a dozen or so molecules, which still allows for the formation of stable water solutions. The reduced form of MB, denoted as MBp, is charge-neutral and, as seen in Figure S3, forms a single aggregate in water. The size of this aggregate is likely limited by the availability of MB molecules in the simulation box.

Considering the mobility of MB molecules in the adsorbed state on the discussed surfaces, diffusivities were determined. The approach applied for this purpose was based on the Einstein relation; thus, diffusivities were calculated as the slopes of the mean square displacement versus time for MB molecules. Table 1 shows the diffusion coefficients for MB molecules in all studied cases, as well as the single-molecule diffusivities for comparison. From these results, we conclude that the formation of clusters or aggregates in bulk solution, as shown in Figure S3, significantly reduces the mobility of dye molecules compared to single-molecule diffusivities. Adsorption on flat graphite surfaces leads to diffusivity values similar to those in clusters; however, it is observed that, for charged MBn molecules, mobility in the adsorbed state is slightly higher than in the ribbon-like clusters in bulk solution. This is due to electrostatic repulsion in the adsorbed layer and the loss of π–π interactions in the ribbon-like clusters. The uncharged MBp molecules exhibit only slightly lower mobility on the flat graphite surface than in the aggregate, due to persistent strong dispersion interactions in the 2D configuration.

On functionalized surfaces, we observe a reduction in the overall diffusivity of adsorbed dye molecules. However, this reduction is minor for MBn molecules on the unidirectionally shocked functionalized surface (compare nG and nZ) and slightly greater for MBp molecules (compare pG and pZ). This indicates that the mobility of adsorbed dye molecules is relatively high, and the adsorption of MB on graphitized surfaces should be considered mobile rather than localized. However, on surfaces with strong corrugation after isotropic compression, diffusivities are significantly reduced (nI and pI), indicating that the adsorbed molecules become immobilized.

**Figure 5:**
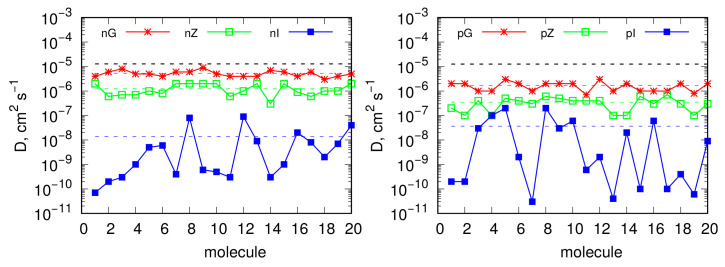
Diffusivities of MB for individual molecules in all studied adsorbed states (points). The colored dashed lines indicate average diffusivities for all molecules, as shown in Table 1. The black dashed lines represent single-molecule diffusivities in bulk water.

Figure 5 allows us to draw more detailed conclusions regarding the state of MB molecules on the studied surfaces. It shows the contributions of individual molecules to the average diffusivities presented in Table 1. We can observe that intact flat graphite surfaces are completely uniform in terms of diffusivity for each MB molecule. Similarly, the functionalized but nearly flat surfaces (nZ and pZ) also show an almost uniform distribution of diffusivities, though with slightly greater dispersion. Generally, the reduction in average diffusivity for flat but functionalized surfaces, compared to ideal graphite surfaces, suggests that interactions with surface functional groups affect molecule mobility.

The substantial variation in diffusivities observed for individual molecules on highly corrugated surfaces (i.e., the nI and pI systems), along with the extreme reduction in diffusivity for some molecules (decreasing by up to 5 orders of magnitude), indicate that these molecules are trapped in an immobile state, while others still display significant mobility. This is due to the limited number of locations on the surface exhibiting such strong interactions with dye molecules. These areas are primarily the bottoms of wells formed on the surfaces, while regions with higher mobility correspond to the slopes of these wells. These conclusions are also supported by a visual analysis of the MD trajectories.

**Figure 6:**
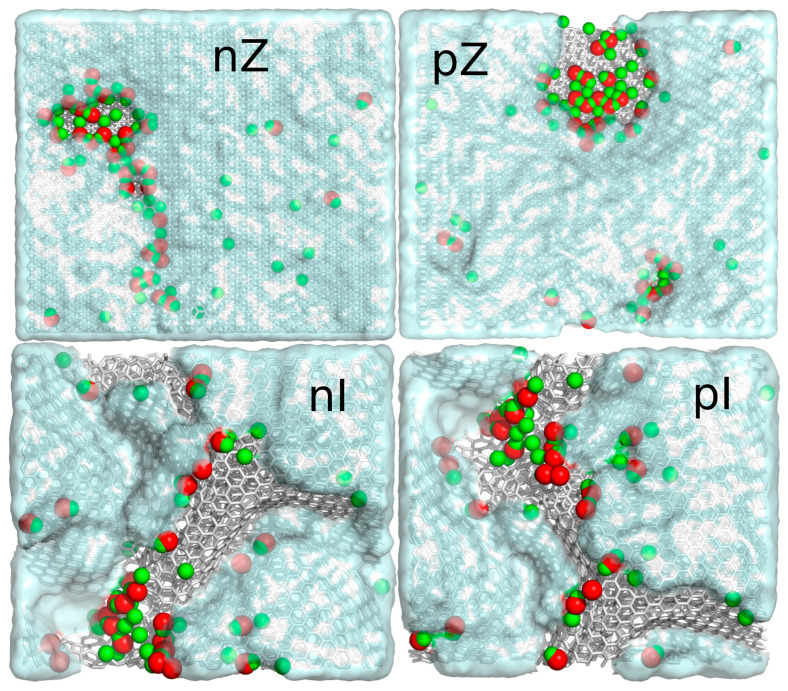
Projections of MB molecules onto static reference surfaces, compiled from all simulation frames. The color codes for the surfaces are the same as in Figure 1, and the projections are represented by semi-transparent cyan clouds. The areas not covered by clouds were never visited by any atom of the MB molecules.

The observed reduction in diffusivities from intact graphite surfaces (nG and pG) to functionalized but flat surfaces (nZ and pZ) suggests that surface hydroxyl groups interact strongly with MB molecules, thereby reducing their mobility. However, a closer inspection of the dynamic trajectories indicates that MB molecules, whether ionized or charge-neutral, tend to avoid areas with surface hydroxyl groups. This can be more effectively visualized by projecting all MB molecules from all simulation frames onto a static reference surface. Such a visualization is shown in Figure 6 for the nZ, pZ, nI, and pI systems on a single surface facet.

As seen in Figure 6, there are areas with surface hydroxyl groups that were never visited by any atom from the MB molecules, even on the flat surfaces of the nZ and pZ systems. This effect cannot be attributed to specific repulsion between surface hydroxyl groups and MB molecules. Although surface hydroxyls carry partial charges, they are effectively charge neutral at a certain distance and do not significantly contribute to surface-adsorbate forces, at least for the charge-neutral MBp molecules. Additionally, the Coulomb components of pair interaction energies shown in Table 1 are small in all cases and not decisive. The most reasonable explanation for this effect is the stronger interaction between water molecules and surface hydroxyls, which prevents MB molecules from occupying these areas due to steric repulsion with the water molecules. In the case of the nI and pI systems, the never-occupied areas are also due to the immobilization of MB molecules at the bottoms of surface valleys. Here, the Lennard–Jones components of pair energies are enhanced, and additionally, they result in slightly positive Coulomb components, as the entrapped molecules may be in less favorable positions for electrostatic interactions.

In generalizing the above observations, we can state that MB molecules on flat graphitized carbon surfaces, such as Sterling FT carbon black, align with the model of mobile adsorption on an almost energetically homogeneous surface. This is because the diffusivities of MB molecules adsorbed on such surfaces are high, and the molecules do not encounter areas with a high density of hydroxyl groups, which could alter the adsorption energy and introduce energetic heterogeneity. On the other hand, surfaces with significant geometric irregularities, such as LMA10, constrain dye molecules to remain at the bottoms of surface valleys. Thus, the adsorption energy aligns with the model of localized adsorption with energetic heterogeneity, as the curvature of the surface valleys varies and becomes the primary factor modifying the adsorption energy.

Thus, the widely used adsorption models, such as Langmuir, Freundlich, and Temkin, [35,36] routinely applied for fitting adsorption data [37,38] from activated carbon surfaces, have a limited fundamental basis, as they are derived by assuming localized adsorption on energetically uniform (Langmuir) or heterogeneous (Freundlich and Temkin) surfaces. A more appropriate model, particularly for graphitized carbon surfaces, would be the Volmer isotherm, [35] which is derived for mobile adsorption. However, the Volmer isotherm was as follows:

(1) $$Kc=\frac{\theta }{1-\theta }exp\left(\frac{\theta }{1-\theta }\right)$$

where *c* is the adsorbate concentration, θ is the surface coverage, and *K* is a constant; therefore, it cannot be linearized, which limits its routine application for dataset analysis. However, differences in the fitting quality of various adsorption isotherms to actual data are usually so small that identifying the true adsorption mechanism based on the fit quality of an adsorption isotherm is quite difficult.

### 2.3. Free Energy of MB Desorption

The free energy of desorption is a thermodynamic indicator of adsorption strength and, as such, is one of the most valuable quantities obtainable from atomistic simulations. The umbrella sampling (US) method allows for the determination of potentials of mean force (PMF), from which binding energies can be estimated. However, umbrella sampling has certain limitations, as it does not allow for the simultaneous determination of the PMF profile for all adsorbed molecules. Effective convergence with this method is achieved when only a single molecule is detached from the adsorbed state in a single run. Therefore, the first step in the US method involves selecting a representative molecule from a given molecular configuration.

**Figure 7:**
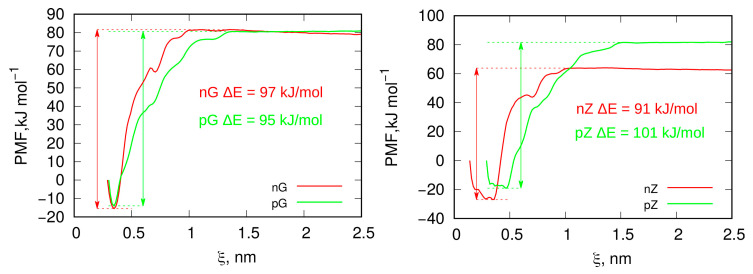
Potentials of mean force (PMF) obtained using umbrella sampling simulations and the weighted histogram analysis method (WHAM) protocol for the desorption of an MB probe molecule from an intact graphite surface (left panel) and from a functionalized graphitized carbon surface generated by unidirectional shock compression. The ΔE values represent the binding energies, determined as the difference between the maximum and minimum PMF values.

For systems with flat or nearly flat geometries, as observed in the previous section, the MB molecules form a 2D mobile phase, meaning that all molecules are in a very similar energetic state. This makes the choice of a molecule for detachment in the US method effectively arbitrary. Consequently, in Figure 7, we analyze the PMF profiles for both surfaces—an intact graphite surface and one subjected to unidirectional shock compression—using a freely chosen MB molecule as a probe for the US simulations.

The PMF profiles represent the effective work required to move the molecule to a specified distance, represented here as the collective variable ξ, which denotes the distance between the center of mass of the dragged molecule and a subset of surface atoms. The variable ξ is one-dimensional and varies only in the z direction. Therefore, the minimum PMF value corresponds to the initial distance when the molecule is in its equilibrium state on the surface. Any change in ξ from this PMF minimum indicates the movement of the probe molecule either toward or away from the surface.

Analyzing the binding energy values—calculated as the difference between the PMF at the desorbed state (highest PMF value) and the adsorbed state (PMF minimum)—leads to the conclusion that adsorption is strong in every combination of surface and MB state. Differences in ΔE values on the order of a few kJ mol^−1^ are likely not significant, given the inherent accuracy limitations of any free energy determination method, including umbrella sampling (US). However, the notably higher ΔE value observed in the pZ system correlates with the considerably lower diffusivity of MB molecules in this system, as shown in Table 1 and Figure 5. This suggests that the reduced mobility of charge-neutral MB molecules in the pZ system, along with the higher binding energy, may be related to lateral interactions between MB molecules.

The general conclusion is that the adsorption of MB molecules in slit-like mesopores of graphitic or graphitized carbon is strong and essentially irreversible under ambient conditions, as the binding energy on the order of 90–100 kJ mol^−1^ makes desorption highly unlikely.

**Figure 8:**
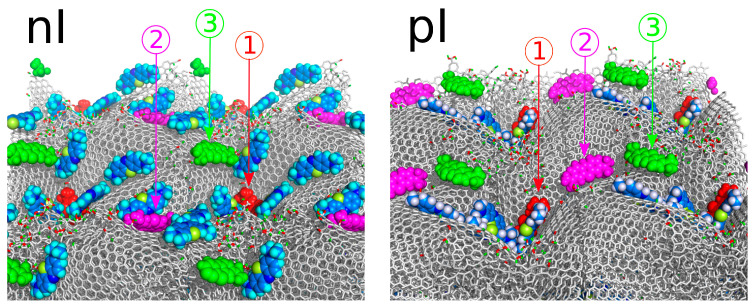
Views of the adsorbing surfaces in a supercell representation, i.e., with replication of the simulation cell in the x and y directions to better visualize the surface geometry and the localization of MB molecules. The numbers (1), (2), and (3) designate the molecules selected for umbrella sampling to determine their binding energies at these specific positions.

The analysis of MB binding on surfaces obtained through isotropic compression, which reveals geometric irregularities, is more complex, as the binding energy will depend on the specific molecule selected or, more precisely, its surrounding environment. To assess the energetic state of molecules adsorbed on morphologically disordered surfaces like LMA10, we identified three representative cases of adsorbed molecule states for both ionized MBn molecules and charge-neutral MBp molecules. These states are numbered from 1 to 3 in Figure 8 to facilitate further discussion.

Figure 8 presents the surface of isotropically shocked graphite in a supercell representation (replicated in the x and y directions) to more clearly show the presence of valleys of varying depths and shapes, along with the localization of dye molecules in these regions. State (1) in both nI and pI systems, marked by a red-colored molecule, corresponds to the deepest valleys on the surface. As seen, the dye molecules fully embed themselves in these pockets. Additionally, these areas are densely occupied, accommodating as many dye molecules as the space allows. State (2), represented by magenta molecules, corresponds to shallower valleys, while state (3), represented by green molecules, is chosen to represent dye molecules adsorbed atop the hills formed between the valleys.

**Figure 9:**
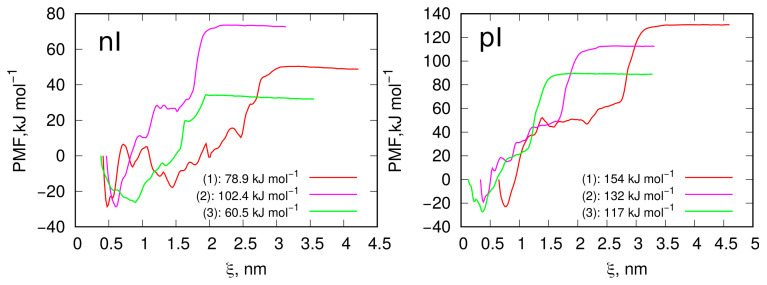
Potentials of mean force (PMF) obtained using umbrella sampling simulations and the weighted histogram analysis method (WHAM) protocol for the desorption of MB probe molecules from various states shown in Figure 8. The energies shown in the figure legends represent binding energies, calculated as the difference between the highest and lowest PMF values for each profile. The collective variable ξ represents the distance between the center of mass of the molecules and the surface atoms located at the bottom, from which detachment was initiated.

The PMF profiles for the detachment of these molecules from the surfaces, obtained using the umbrella sampling technique, are shown in Figure 9, along with binding energies, determined as the difference between the highest and lowest PMF values for each profile. As seen, the PMF profiles are more complex compared to those from flat surfaces, with multiple intermediate states displaying local energy minima. This complexity is expected, as during detachment from very deep valleys the molecule may jump to adjacent valleys and become temporarily trapped until the pulling force detaches it again.

However, the determined overall binding energies, shown in the figure legends, reveal some unexpected relationships. Most surprising is the lower binding energy of the ionized MBn molecule in the deepest valley (state 1), compared to that on the flat surface (see Figure 7). Intuitively, state (1) in Figure 9 for the nI system should correspond to a stronger binding energy than on a flat surface; however, this is not the case. A possible explanation for this is either fewer close contacts on the corrugated surface or an effect of electrostatic repulsion with other molecules in the same valley (with three neighboring molecules present within the same valley).

State (2) corresponds to a gentler surface curvature, allowing more close contacts to form, while only two other MBn molecules are in the close vicinity of the probe molecule. This results in a binding energy comparable to or slightly greater than that on flat surfaces. Finally, state (3) yields, as expected, the lowest binding energy, as the probe molecule sits on a convex surface area with only one neighboring MBn molecule.

The charge-neutral MBp molecules, however, exhibit significantly stronger binding on corrugated surfaces compared to flat ones, in contrast to MBn molecules. The binding energy in the deepest valley (state 1) reaches 154 kJ mol^−1^, which is 150% of the binding energy on flat surfaces. This increase, and the reverse trend compared to the nI system in state (1), likely results from attractive dispersion interactions between MBp molecules and the absence of electrostatic repulsion. In state (1), three other MBp molecules are adsorbed within the same valley as the probe molecule, resulting in substantial nearest neighbor interactions that contribute significantly to the binding energy.

In state (2), the binding energy is lower but still higher than on a flat surface, due to a larger number of close contacts with surface atoms in this configuration. Finally, state (3) has the smallest binding energy, though it remains greater than on a flat surface. In this state, the probe molecule is relatively mobile, frequently moving to neighboring molecules and positioning itself on the slopes of the hill. Consequently, its effective binding energy lies between that of the flat surface and state (2), where the molecule settles into a surface pocket, increasing the number of close contacts with the surface.

## 3. Materials and Methods

### 3.1. Preparation of Initial Graphite Slab

The initial structure of the graphite slab was constructed manually using standard lattice parameters. The slab had an initial horizontal dimension of 5.883 nm × 6.794 nm and consisted of 6 graphene layers to ensure sufficient thickness, preventing pairwise interactions between atoms on the upper and lower surfaces of the slab. This graphite slab was subjected to relaxation simulations using the standard pairwise additive general Amber force field (gaff2) [39,40]. The force field topology files were generated using the tleap program from the AmberTools22 package [41] and AcPyPE script [42]. The computations devoted to preparing the solid substrate were carried out using the LAMMPS code [43]. Specifically, the initial steps were performed using simulations in the isotropic NPT ensemble, which allowed for the horizontal relaxation of the graphite slab. The conditions were standard—a temperature of 300 K and a pressure of 1 bar, with an integration timestep of 0.002 fs. This prepared solid substrate was used either in subsequent simulations of activation of shock compression in water media, or simply in direct adsorption of methylene blue, serving as a reference state for the activated surfaces.

### 3.2. Shock Compression

Activation of the graphite surface was carried out using shock compression in the presence of water. Shock compression in molecular simulations is a powerful method for studying the behavior of materials under extreme pressure and temperature conditions, such as those encountered during impacts, explosions, or in planetary interiors. The principles underlying shock compression involve understanding how a material’s structure, dynamics, and thermodynamic properties respond when subjected to a sudden, high-pressure shock wave.

In molecular simulations, these principles are implemented to investigate atomic-level phenomena that are difficult to observe experimentally. The foundation of shock compression is based on the Rankine–Hugoniot relations, [44] which are derived from the conservation of mass, momentum, and energy across a shock front. These relations describe the state of a material after shock compression by linking initial and final states, such as pressure, density, and internal energy.

The mass flux must remain constant across the shock wave:

(2) $$\rho_{0}U_{s}=\rho_{1}\left(U_{s}-u_{p}\right)$$

where *ρ*_0_ and *ρ*_1_ are the initial and final densities and *U_s_* and *u_p_* are the shock wave and particle velocities, respectively. The momentum change is balanced by the pressure difference:

(3) $$P_{1}-P_{0}=\rho_{0}U_{s}u_{p}$$

where *P*_0_ and *P*_1_ are the initial and final pressures. The energy change is determined by the work carried out by the shock wave:

(4) $$E_{1}-E_{0}=\frac{1}{2}\left(P_{0}+P_{1}\right)\left(V_{0}-V_{1}\right)$$

where *E*_0_ and *E*_1_ are the initial and final energies. These equations provide a theoretical framework to predict how a material will respond to a shock wave and serve as boundary conditions in molecular simulations. In our particular case, we are primarily interested in possible chemical transformations within the material induced by shock waves of various magnitudes.

In simulations, shock waves are typically generated using two common methods:

- Direct Impact Method: A moving slab or piston is introduced into the simulation domain to physically drive a shock wave through the material;
- Hugoniostat Method: Instead of directly inducing a shock wave, the simulation adjusts the system parameters to satisfy the Hugoniot conditions, creating a virtual shock that corresponds to a particular pressure and temperature state [23].

The Hugoniostat method [23], implemented in LAMMPS as fix nphug, was used in this study to initiate reactions between graphite and water and to simulate deformations of the bulk material. Depending on the settings, this procedure allows for the simulation of shock waves acting in one direction, or isotropically in all principal axes. This leads to significant differences in the resulting material morphology and chemical composition. The essential results and differences between the unshocked sample, the sample after shock compression in the z direction only, and the sample shocked in the x, y, and z directions are outlined in Figure 1. Simulation settings used in the shock compression computations can be found in the LAMMPS input scripts available in the supplementary information file.

### 3.3. Reactive Force Field

Reactions between water and carbon atoms can occur due to the application of a reactive force field for molecular dynamics, that is, the ReaxFF force field [28,45]. The functional form of the ReaxFF force field is highly versatile, as it incorporates a bond order-dependent formalism to handle the dynamic formation and breaking of bonds in simulations. It includes terms for bond energy, angle, and torsion interactions, van der Waals forces, Coulomb interactions (using a charge equilibration method), and specific penalty functions to account for the over-coordination or under-coordination of atoms. This allows ReaxFF to accurately capture the energetics of both covalent and ionic systems, making it suitable for a wide range of chemical environments [21].

Parameterization in ReaxFF involves fitting the force field parameters to quantum mechanical (QM) data or experimental observations for a specific system. This fitting process is crucial for ensuring that the model accurately represents the behavior of materials in both equilibrium and reactive states. Parameters include bond lengths, bond strengths, angles, and van der Waals radii, and they are often system-specific, requiring extensive training against high-level QM data for each element or material of interest. In this work, we utilized the recent parameterization of the ReaxFF force field for the oxidation and pyrolysis of hydrocarbon fuels published by Ashraf and van Duin [28]. The force field file containing all the parameter values is available in the supplementary file.

### 3.4. Methylene Blue

**Figure 10:**
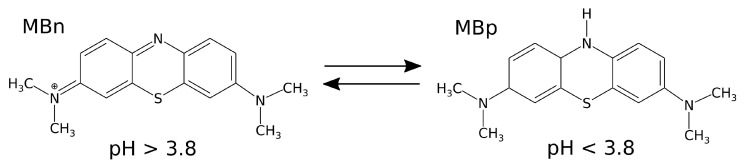
Structural formulas of the ionized and neutral forms of Methylene Blue.

Methylene Blue is considered in two forms: the ionized (oxidized) form, MBn, which exists at pH > 3.8, and the neutral (reduced) form, MBp, which is typical at pH < 3.8 [6,38]. Its structural formulas are shown in Figure 10.

The force field for MB simulations was based on the general Amber force field (gaff2), generated using the AmberTools22 package and the AcPyPE script. The partial charges for both forms of MB were obtained using the RESP (Restrained Electrostatic Potential) procedure [46,47].

### 3.5. Computational Details

The simulation boxes were constructed by forming slit-like pores using the activated carbon slabs and applying periodic boundary conditions in the z direction. The pore widths were 5 nm for the intact graphite slab, between 5.1 and 5.6 nm for the slab after unidirectional shock compression, and between 8.5 and 8.9 nm for isotropic compression. These pore widths for surfaces after shock compression are not precisely defined, due to their corrugated nature. However, in all cases, the pore widths are sufficiently large to ensure that there are no interactions between surface atoms and molecules adsorbed on these surfaces.

In the space between the surfaces, 20 Methylene Blue molecules, appropriate amounts of Cl^−^ ions, and TIP3P water molecules were inserted using custom-designed computer codes. This number of MB molecules was sufficient to study the behavior of the adsorbed layer, and corresponded to a relatively high dye concentration in water, approximately 0.2 mol L^−1^. The number of water molecules was around 6000, with slight variations between systems. The calculations were performed using the GROMACS [21] software with GPU acceleration, consisting of equilibration and production runs, each lasting 80 ns of simulation time, with all other settings kept at their typical values for biomolecular systems.

The potentials of mean force (PMF) for MB desorption from surfaces were determined using the umbrella sampling method and the GROMACS pull code, in combination with the gmx WHAM procedure [48] for histogram reweighting and PMF recovery. Each umbrella window corresponded to a 20 ns trajectory with a force constant of 1000 kJ mol^−1^nm^−2^, and successive windows were separated by 0.2 nm or less.

## 4. Summary and Conclusions

This study investigates the formation and functionalization of carbon surfaces under shock compression using molecular dynamics simulations with the ReaxFF reactive force field. By applying controlled shock waves to graphite in a water environment, the study successfully creates functionalized and morphologically varied carbon surfaces. The compression approaches used—unidirectional and isotropic—result in distinct surface morphologies and chemical characteristics, with the former preserving a relatively flat structure, while the latter generates a corrugated surface with deep valleys and high ridges. These changes have significant implications for surface properties, which were examined through adsorption studies of methylene blue (MB) in both ionized and neutral forms.

The adsorption simulations reveal that MB molecules interact differently with the variously structured carbon surfaces. On flat surfaces, MB retains high mobility, whereas on corrugated surfaces—especially in the deepest valleys—MB molecules tend to be immobilized. This behavior has significant implications for commonly used approaches to analyzing adsorption isotherms and adsorption kinetics on activated carbons. Standard isotherms, such as Langmuir, Temkin, and Freundlich, are derived from localized adsorption models, assuming either energetically uniform or heterogeneous surfaces. However, simulation data suggest that MB adsorption on graphitized carbons without geometric corrugation is both strong and mobile, thus not meeting the assumptions of these adsorption isotherms.

The properties of the adsorbate, in this case MB, are also predictable. Flat molecules, such as bisazo dyes, phenothiazine dyes, or other compounds with multiple aromatic rings, tend to lie flat on graphene-like surfaces and exhibit lateral mobility across the surface. Only significant geometric corrugation results in the immobilization of adsorbate molecules within valleys, where a high adsorption binding energy is not required to keep them in place. However, this binding energy varies depending on the shape and depth of the valleys, their availability, and interactions with neighboring adsorbate molecules. Consequently, the Langmuir isotherm is not formally appropriate for analyzing adsorption data from such corrugated surfaces.

The role of surface patches highly functionalized with hydroxyl groups proved intriguing, as the adsorbate molecules consistently avoided these areas throughout the entire simulation period. This behavior can be explained by the preferential occupation of these functionalized areas by water molecules, thereby limiting access for MB molecules. From this, we can draw an additional conclusion regarding highly oxidized activated carbons and adsorption in aqueous environments; water may block regions with a high density of hydroxyl groups, reducing access to these areas for adsorbate molecules. However, this effect likely depends on the type of adsorbate molecule, as some molecules may outcompete water and preferentially adsorb onto such areas. For MB, however, we observed the clear avoidance of these highly hydroxylated regions by the adsorbate molecules.

The adsorption binding energy of MB is uniform on graphitized surfaces, and high (90–100 kJ mol^−1^) for both forms of MB. The adsorption on corrugated surfaces reveals diverse behavior for MBn and MBp. In both cases, the adsorption was stable and localized, but the binding energy for charged molecules (MBn) was reduced in the valleys (or generally in narrow micropores) due to electrostatic repulsion. Conversely, the binding energy of charge-neutral MBp molecules was enhanced by 150% in the deep valleys. All this means that carbon surfaces should be very effective in removing this type of dye from water solutions.

## Figures and Tables

**Table 1:** Diffusion coefficients of MB molecules in various cases: single-particle diffusion in water, cluster diffusion in water (Figure S3), and the average diffusion of MB molecules adsorbed on the discussed surfaces. The pair energies refer to the Lennard–Jones and electrostatic components of MB interactions with the surfaces per single MB molecule.

	Diffusivity, 10^−5^ cm^2^ s^−1^	Pair Energy, kJ mol^−1^	
		Lennard–Jones	Coulomb
single MBn molecule	1.279		
single MBp molecule	1.256		
20 MBn molecules, Figure S3	0.359		
20 MBp molecules, Figure S3	0.299		
nG system	0.530	−172	−1.17
nZ system	0.122	−173	−2.35
nI system	0.00137	−199	+11.3
pG system	0.170	−172	−0.52
pZ system	0.0348	−172	−2.80
pI system	0.00365	−200	+0.75

## Data Availability

The data are available from the authors upon request.

## Supplementary Materials

The following supporting information can be downloaded at: https://www.mdpi.com/article/10.3390/molecules29246030/s1. Figure S1: Zoomed-in areas of the extensively functionalized graphite surface after unidirectional shock compression in the direction normal to the surface are shown. Red and green sticks correspond to oxygen and hydrogen atoms, respectively, while gray sticks represent connections between carbon atoms. Figure S2: Zoomed-in areas of the extensively functionalized graphite surface after isotropic shock compression in the x, y, and z directions are shown. Red and green sticks correspond to oxygen and hydrogen atoms, respectively, while gray sticks represent connections between carbon atoms. Several example carbonyl and ether/epoxy groups are denoted by arrows. Figure S3: Simulation snapshots of the equilibrium structures of MBn and MBp molecules in water (without surfaces) at T = 300 K and p = 1 bar. LAMMPS input scripts for shock compression simulations and parameters of the ReaxFF force field.
