# Supplementary material for: Adsorption of Methylene Blue on Activated Carbon Surfaces Obtained by Shock Compression of Graphite Using Reactive Molecular Dynamics
Source: Molecules. 2024 Dec 21;29(24):6030. doi: 10.3390/molecules29246030 (PMC11679267; doi:10.3390/molecules29246030)
Supplement: Supplementary file 1 [file molecules-29-06030-s001.zip › molecules-3349755-supplementary.pdf]

Adsorption of Methylene Blue on Activated Carbon Surfaces Obtained by Shock Compression of Graphite Using Reactive Molecular Dynamics

Tomasz Panczyk[1,*], Paweł Wolski[1], Krzysztof Nieszporek[2] and Robert Pietrzak[3]

[1]Jerzy Haber Institute of Catalysis and Surface Chemistry, Polish Academy of Sciences ul. Niezapominajek 8,30239 Cracow, Poland
[2]Department of Theoretical Chemistry, Institute of Chemical Sciences, Faculty of Chemistry, Maria Curie-Sklodowska University in Lublin pl. Maria Curie-Sklodowska 3, 20031 Lublin, Poland
[3]Department of Applied Chemistry, Faculty of Chemistry, Adam Mickiewicz University in Poznań, Uniwersytetu Poznańskiego 8, 61-614 Poznań, Poland

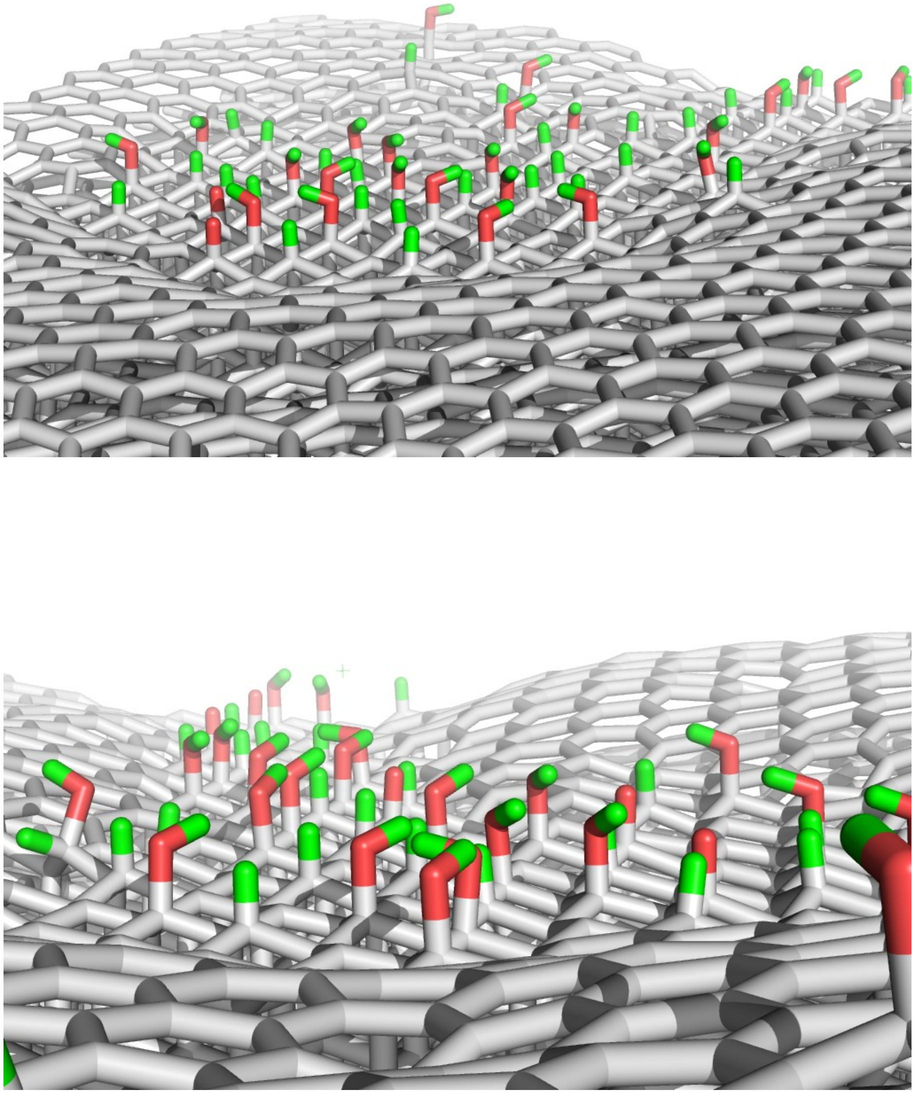

Figure S1. Zoomed-in areas of the extensively functionalized graphite surface after unidirectional shock compression in the direction normal to the surface are shown. Red and green sticks correspond to oxygen and hydrogen atoms, respectively, while gray sticks represent connections between carbon atoms.

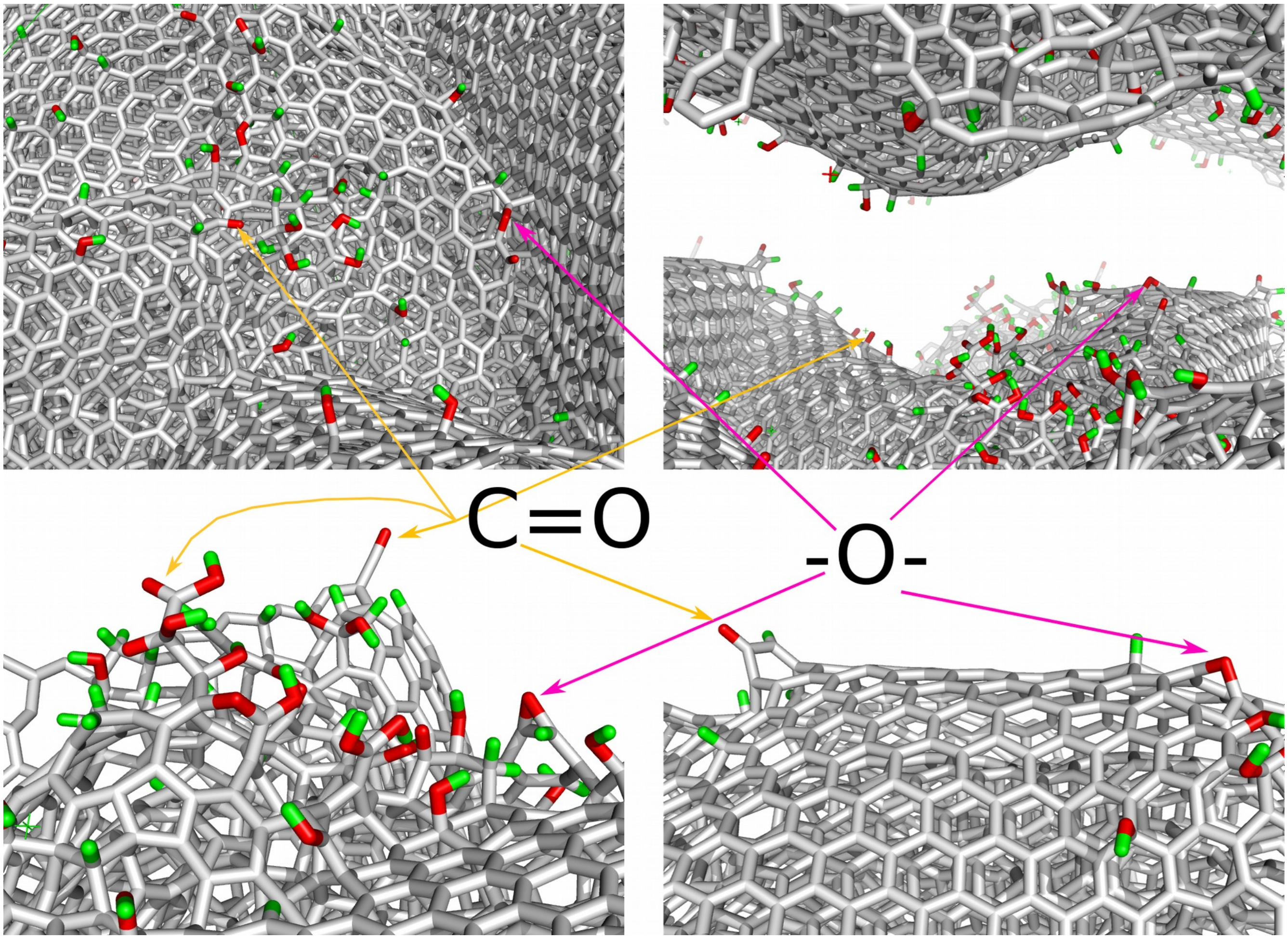

Figure S2. Zoomed-in areas of the extensively functionalized graphite surface after isotropic shock compression in the x, y, and z directions are shown. Red and green sticks correspond to oxygen and hydrogen atoms, respectively, while gray sticks represent connections between carbon atoms. Several example carbonyl and ether/epoxy groups are denoted by arrows.

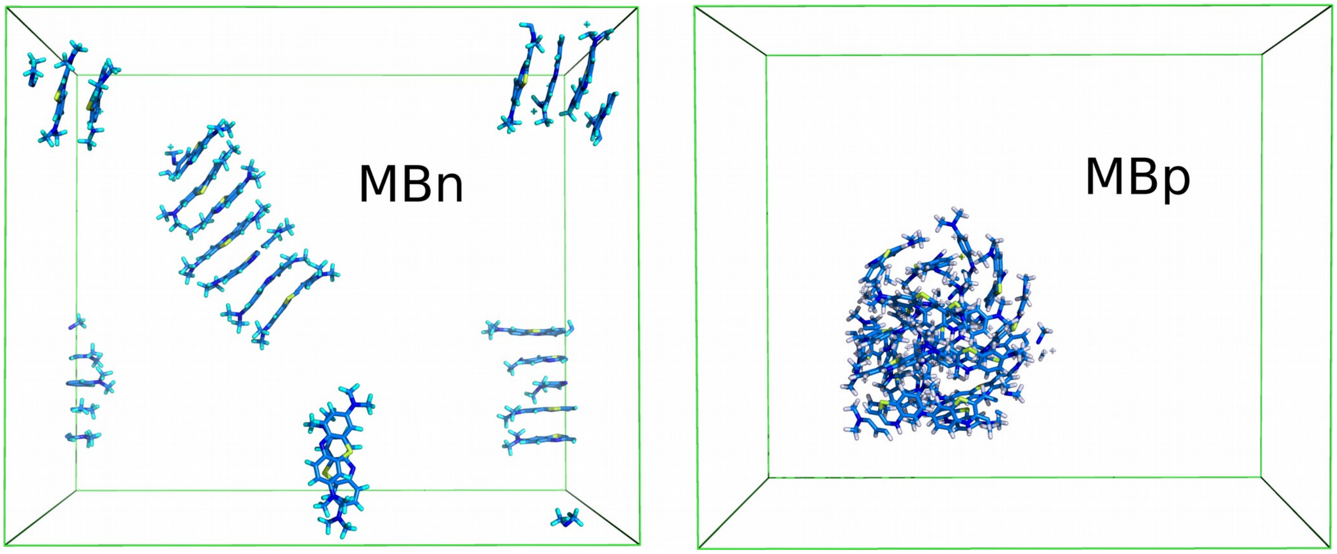

Figure S3. Simulation snapshots of the equilibrium structures of MBn and MBp molecules in water (without surfaces) at T = 300 K and p = 1 bar.

- LAMMPS input script for carrying hugoniot simulations in z direction:

```
#LAMMPS (15 June 2023)
dimension   3
units real
boundary    p p p
atom_style  full
read_data   name.data
timestep    0.250000
pair_style reaxff NULL checkqeq yes lgvdw no safezone 3.0 mincap 200
pair_coeff * * ./ffield_CHO_Ashraf_2017 C O H
fix qeq all qeq/reaxff 1 0.0 10.0 1.0e-6 reaxff
fix bonds all reaxff/bonds 10000 name.bonds.gz
fix species all reaxff/species 10 1 1000 name.species element C O H
fix int all nphug temp 300.000000 300.000000 50.000000 z 4e5 4e5 2250.000000
run   200000
```

- LAMMPS input script for carrying hugoniot simulations in all directions (xyz):

```
#LAMMPS (15 June 2023)
dimension   3
units real
boundary    p p p
atom_style  full
read_data   name.data
timestep    0.250000
pair_style reaxff NULL checkqeq yes lgvdw no safezone 3.0 mincap 200
pair_coeff * * ./ffield_CHO_Ashraf_2017 C O H
fix qeq all qeq/reaxff 1 0.0 10.0 1.0e-6 reaxff
fix bonds all reaxff/bonds 10000 name.bonds.gz
fix species all reaxff/species 10 1 1000 name.species element C O H
fix int all nphug temp 300.000000 300.000000 50.000000 iso 4e5 4e5 2250.000000
run   200000
```

- ReaxFF force field parameters utilized in this study (Ashraf, C.; van Duin, A.C.T. Extension of the ReaxFF Combustion Force Field toward Syngas Combustion and Initial Oxidation Kinetics. *J. Phys. Chem. A* **2017**, *121*, 1051–1068, doi:10.1021/acs.jpca.6b12429)

```
Content of ffield_CHO_Ashraf_2017 file:
Reactive MD-force field: Jul21 2016 Ashraf c/h/o
 39       ! Number of general parameters
   50.0000 !p(boc1)
    9.5469 !p(boc2)
   26.5405 !p(coa2)
    0.6863 !p(trip4)
    2.7295 !p(trip3)
   70.0000 !kc2
    1.0588 !p(ovun6)
    4.1262 !p(trip2)
   12.1176 !p(ovun7)
   13.3056 !p(ovun8)
  -68.9784 !p(trip1)
    0.0000 !Lower Taper-radius (swa)
   10.0000 !Upper Taper-radius (swb)
    0.0000 !not used
   33.8667 !p(val7)
    6.0891 !p(lp1)
    1.0563 !p(val9)
```

```
   2.0384 !p(val10)
   6.1431 !not used
   6.9290 !p(pen2)
   0.3989 !p(pen3)
   3.9954 !p(pen4)
   0.0000 !not used
   5.7796 !p(tor2)
  10.0000 !p(tor3)
   1.9487 !p(tor4)
   0.0000 !not used
   2.1645 !p(cot2)
   1.5591 !p(vdW1)
   0.1000 !Cutoff for bond order*100 (cutoff)
   2.1365 !p(coa4)
   0.6991 !p(ovun4)
  50.0000 !p(ovun3)
   1.8512 !p(val8)
   0.0000 !not used
   0.0000 !not used
   0.0000 !not used
   1.0000 !not used
   2.6962 !p(coa3)
11    ! Nr of atoms; atomID;ro(sigma); Val;atom mass;Rvdw;Dij;gamma
           alfa;gamma(w);Val(angle);p(ovun5);n.u.;chiEEM;etaEEM;n.u.
           ro(pipi);p(lp2);Heat increment;p(boc4);p(boc3);p(boc5),n.u.;n.u.
           p(ovun2);p(val3);n.u.;Val(boc);p(val5);n.u.;n.u.;n.u.
C    1.3674   4.0000  12.0000   2.0453   0.1444   0.9500   1.1706   4.0000
     9.0000   1.5000   4.0000  27.5134  79.5548   5.0191   7.0500   0.0000
     1.1168   0.0000 181.0000  14.2732  24.4406   6.7313   0.8563   0.0000
    -4.1021   5.0000   1.0564   4.0000   2.9663   0.0000   0.0000   0.0000
H    0.9479   1.0000   1.0080   1.1364   0.0232   0.9900  -0.1000   1.0000
     9.0643   4.7746   1.0000   0.0000 121.1250   4.7757   9.7732   1.0000
    -0.1000   0.0000  62.4879   2.5194   2.3785   0.2223   1.0698   0.0000
   -15.7683   2.1488   1.0338   1.0000   2.8793   0.0000   0.0000   0.0000
O    1.1939   2.0000  15.9990   1.9289   0.1201   0.9900   1.0981   6.0000
    10.4842   8.2916   4.0000  28.8967 116.0768   7.9703   7.0500   2.0000
     1.0479  20.0000  60.8726  10.0338   2.2024   0.9942   0.9745   0.0000
    -3.6141   2.7025   1.0493   4.0000   2.9225   0.0000   0.0000   0.0000
S    1.9186   2.0000  32.0600   1.6516   0.4937   0.7530   1.6593   6.0000
     9.0227   4.9055   4.0000  30.0000 112.1416   6.5745   9.0000   2.0000
     1.0000   3.4994  65.0000  12.0000  22.1978  15.3230   0.9745   0.0000
   -15.7363   2.8802   1.0338   6.2998   2.8793   0.0000   0.0000   0.0000
Mo   2.4695   5.6375  95.9400   1.8471   0.3413   1.0000   0.1000   6.0000
    13.1958  44.8826   4.0000   0.0000   0.0000   0.7695   6.0677   0.0000
     0.1000   0.0000 152.6300   3.4529   0.0722   3.1767   0.8563   0.0000
   -17.9815   3.1072   1.0338   8.0000   3.4590   0.0000   0.0000   0.0000
Ni   1.8201   2.0000  58.6900   1.9449   0.1880   0.8218   0.1000   2.0000
    12.1594   3.8387   2.0000   0.0000   0.0000   4.8038   7.3852   0.0000
    -1.0000   0.0000  95.6300  50.6786   0.6762   0.0981   0.8563   0.0000
    -3.7733   3.6035   1.0338   8.0000   2.5791   0.0000   0.0000   0.0000
Li   1.9814   1.0000   6.9410   1.8000   0.2939   0.9387  -0.1000   1.0000
     9.0616   1.3258   1.0000   0.0000   0.0000  -3.0000  10.0241   0.0000
    -1.0000   0.0000  37.5000   5.4409   6.9107   0.1973   0.8563   0.0000
    -2.5068   2.2989   1.0338   1.0000   2.8103   1.3000   0.2000  13.0000
B    1.5530   3.0000  10.8110   1.6512   0.1000   0.9480   1.0000   3.0000
    10.3025   2.3647   3.0000   0.7036  80.0000   4.0000   7.0000   0.0000
    -1.3000   0.0000 151.3700   7.6069   1.9324   1.0943   0.0000   0.0000
    -3.1611   4.0000   1.0564   3.0000   2.8413   0.0000   0.0000   0.0000
F    1.1846   1.0000  18.9984   1.7922   0.1267   0.5000  -0.1000   7.0000
    10.3184   7.5000   1.0000   9.2533   0.2000   9.0000   8.0000   0.0000
    -1.0000  35.0000  18.0000   6.9821   4.1799   1.0561   0.0000   0.0000
    -7.3000   2.6656   1.0493   4.0000   2.9225   0.0000   0.0000   0.0000
P    1.5994   3.0000  30.9738   1.7000   0.1743   1.0000   1.3000   5.0000
     9.1909  14.9482   5.0000   0.0000   0.0000   1.8000   7.0946   0.0000
    -1.0000  25.0000   1.5000   0.2187  21.4305  15.1425   0.0000   0.0000
    -3.9294   3.4831   1.0338   5.0000   2.8793   0.0000   0.0000   0.0000
X   -0.1000   2.0000   1.0080   2.0000   0.0000   1.0000  -0.1000   6.0000
    10.0000   2.5000   4.0000   0.0000   0.0000   8.5000 999.5000   0.0000
    -0.1000   0.0000 127.6226   8.7410  13.3640   0.6690   0.9745   0.0000
   -11.0000   2.7466   1.0338   6.2998   2.8793   0.0000   0.0000   0.0000
43      ! Nr of bonds; at1;at2;De(sigma);De(pi);De(pipi);p(be1);p(b
                      p(be2);p(bo3);p(bo4);n.u.;p(bo1);p(bo2)
  1  1  80.8865 107.9944  52.0636   0.5218  -0.3636   1.0000  34.9876   0.7769
         6.1244  -0.1693   8.0804   1.0000  -0.0586   8.1850   1.0000   0.0000
```

1	2	179.5195	0.0000	0.0000	-0.5242	0.0000	1.0000	6.0000	0.7187
		5.4740	1.0000	0.0000	1.0000	-0.1144	6.7029	0.0000	0.0000
2	2	113.9232	0.0000	0.0000	-0.5971	0.0000	1.0000	6.0000	0.9093
		1.7152	1.0000	0.0000	1.0000	-0.0450	6.0710	0.0000	0.0000
1	3	136.4945	164.1201	5.5000	-0.9159	-0.1075	1.0000	10.6519	0.8644
		0.6858	-0.4602	9.5754	1.0000	-0.1745	4.5987	0.0000	0.0000
3	3	148.0798	155.2406	20.1160	-1.0000	-0.1254	1.0000	33.0027	0.7790
		0.7673	-0.1697	7.0028	1.0000	-0.1300	5.1959	1.0000	0.0000
2	3	169.1351	0.0000	0.0000	-0.8810	0.0000	1.0000	6.0000	0.5757
		1.5482	1.0000	0.0000	1.0000	-0.1788	4.6622	0.0000	0.0000
1	4	207.6211	73.7986	55.2528	-0.5497	-0.5211	1.0000	18.9617	0.2217
		1.9950	-0.1000	14.0134	1.0000	-0.1480	5.4471	1.0000	0.0000
2	4	183.1582	0.0000	0.0000	-0.7544	0.0000	1.0000	6.0000	0.3725
		11.7366	1.0000	0.0000	1.0000	-0.0595	4.6177	0.0000	0.0000
4	4	84.3765	31.1563	0.0000	-0.8610	-0.4781	1.0000	17.8574	0.3198
		0.4942	-0.1773	8.4125	1.0000	-0.0889	6.8515	1.0000	0.0000
1	5	0.5356	0.9614	0.0000	0.3817	-0.3000	1.0000	36.0000	0.2142
		0.6116	-0.2579	6.1366	1.0000	-0.0913	6.6008	1.0000	0.0000
2	5	101.0000	0.0000	0.0000	-0.5019	-0.3000	0.0000	36.0000	0.3712
		0.0705	-0.3027	15.0243	1.0000	-0.0950	6.5090	0.0000	0.0000
3	5	108.9868	10.5806	137.5564	0.8861	-0.2172	1.0000	19.1047	1.2087
		0.9510	-0.1831	7.2198	1.0000	-0.1266	6.0906	1.0000	0.0000
4	5	82.5107	27.2572	137.6546	1.0000	-0.2304	1.0000	19.1688	0.4660
		1.0151	-0.1596	7.8950	1.0000	-0.0909	5.5509	1.0000	0.0000
5	5	51.8235	0.0000	0.0000	0.8271	-0.3000	0.0000	16.0000	0.2670
		0.2248	-0.3000	16.0000	1.0000	-0.1908	7.3978	0.0000	0.0000
3	4	145.3431	237.5033	0.0000	0.1826	-0.2406	1.0000	22.1005	0.0500
		0.6769	-0.2612	8.4442	1.0000	-0.1154	6.2859	1.0000	0.0000
1	6	83.5810	9.0383	0.0000	0.2531	-0.2000	1.0000	16.0000	0.0529
		1.4085	-0.1113	13.3900	1.0000	-0.1436	4.5683	1.0000	0.0000
2	6	114.7566	0.0000	0.0000	-0.8939	0.0000	1.0000	6.0000	0.1256
		0.1054	1.0000	0.0000	1.0000	-0.1196	5.0815	0.0000	0.0000
3	6	105.3618	0.0000	0.0000	-0.0456	-0.2000	1.0000	16.0000	0.1870
		0.7193	-0.2500	15.0000	1.0000	-0.0880	5.7169	1.0000	0.0000
6	6	91.2220	0.0000	0.0000	-0.2538	-0.2000	0.0000	16.0000	0.2688
		1.4651	-0.2000	15.0000	1.0000	-0.1435	4.3908	0.0000	0.0000
5	6	56.5379	0.0000	0.0000	-0.3241	-0.2000	0.0000	16.0000	0.1607
		2.6232	-0.2000	15.0000	1.0000	-0.1790	4.4051	0.0000	0.0000
4	6	79.7256	0.0000	0.0000	0.3100	-0.2000	0.0000	16.0000	0.1466
		0.7435	-0.2500	25.0000	1.0000	-0.0929	5.3027	0.0000	0.0000
1	7	54.6610	-0.0200	0.0000	-0.8605	-0.5000	0.0000	35.0000	0.3953
		0.6908	-0.2500	11.9965	1.0000	-0.0668	9.0596	0.0000	0.0000
2	7	59.2034	0.0000	0.0000	0.1240	0.0000	0.0000	6.0000	0.4000
		1.0000	0.0000	12.0000	1.0000	-0.0565	4.9575	0.0000	0.0000
3	7	110.3978	-0.0200	0.0000	-0.1703	0.3000	0.0000	6.0000	0.5194
		0.9844	-0.2500	11.9965	1.0000	-0.0535	7.2629	0.0000	0.0000
4	7	68.4187	0.0000	0.0000	-0.3842	-0.5000	0.0000	25.0000	0.2561
		0.4468	-0.2500	20.0000	1.0000	-0.1990	6.0316	0.0000	0.0000
7	7	34.3154	0.0000	0.0000	0.5995	0.3000	0.0000	26.0000	0.5445
		0.5752	0.0000	12.0000	1.0000	-0.1382	4.5000	0.0000	0.0000
1	8	180.3526	50.0000	0.0000	-0.1860	-0.4591	1.0000	37.7369	0.2590
		0.2807	-0.2047	10.2887	1.0000	-0.0641	5.9561	1.0000	0.0000
2	8	165.3660	0.0000	0.0000	-0.2658	-0.3000	1.0000	25.0000	0.3019
		6.1522	0.0000	0.0000	1.0000	-0.0933	5.4815	1.0000	0.0000
3	8	236.5417	65.2243	0.0000	-0.4987	-0.2500	1.0000	25.0000	1.0000
		0.9994	-0.2342	17.4842	1.0000	-0.1262	5.8863	1.0000	0.0000
4	8	0.0000	0.0000	0.0000	0.9000	-0.2500	1.0000	25.0000	0.5201
		1.0000	-0.1488	10.0786	1.0000	-0.1647	6.3839	1.0000	0.0000
8	8	85.8601	0.0000	0.0000	1.0000	-0.2500	1.0000	25.0000	0.7894
		0.8860	-0.2000	25.0000	1.0000	-0.0820	8.6292	1.0000	0.0000
1	9	204.9977	0.0000	0.0000	-0.8335	-0.5000	1.0000	35.0000	0.3565
		1.5261	-0.2500	15.0000	1.0000	-0.0829	6.1614	1.0000	0.0000
2	9	210.8851	0.0000	0.0000	-0.5848	-0.2000	0.0000	16.0000	0.3016
		3.2045	-0.2000	15.0000	1.0000	-0.0808	6.1810	0.0000	0.0000
3	9	0.0000	0.0000	0.0000	0.2500	-0.5000	1.0000	45.0000	0.6000
		0.4000	-0.2500	15.0000	1.0000	-0.1000	10.0000	1.0000	0.0000
7	9	0.0000	0.0000	0.0000	0.2500	-0.5000	1.0000	45.0000	0.6000
		0.4000	-0.2500	15.0000	1.0000	-0.1000	10.0000	1.0000	0.0000
8	9	150.6978	0.0000	0.0000	0.1373	-0.1418	1.0000	13.1260	0.3916
		0.2867	-0.1310	10.7257	1.0000	-0.1182	6.8737	1.0000	0.0000
9	9	109.0438	0.0000	0.0000	0.6382	-0.3500	1.0000	25.0000	1.1695
		0.1254	-0.2500	15.0000	1.0000	-0.1062	5.9666	1.0000	0.0000
1	10	0.0000	0.0000	0.0000	0.2500	-0.5000	1.0000	45.0000	0.6000
		0.4000	-0.2500	15.0000	1.0000	-0.1000	10.0000	1.0000	0.0000

```
  2 10   0.0000   0.0000   0.0000   0.2500  -0.5000   1.0000  45.0000   0.6000
         0.4000  -0.2500  15.0000   1.0000  -0.1000  10.0000   1.0000   0.0000
  3 10   0.0000   0.0000   0.0000   0.2500  -0.5000   1.0000  45.0000   0.6000
         0.4000  -0.2500  15.0000   1.0000  -0.1000  10.0000   1.0000   0.0000
  7 10   0.0000   0.0000   0.0000   0.2500  -0.5000   1.0000  45.0000   0.6000
         0.4000  -0.2500  15.0000   1.0000  -0.1000  10.0000   1.0000   0.0000
  9 10 153.5200   0.0000   0.0000   0.3010  -0.5000   1.0000  50.0000   0.1025
         0.4150  -0.5000  15.0000   1.0000  -0.0723   5.3872   1.0000   0.0000
 10 10   0.0000   0.0000   0.0000   0.2500  -0.5000   1.0000  45.0000   0.6000
         0.4000  -0.2500  15.0000   1.0000  -0.1000  10.0000   1.0000   0.0000
 28    ! Nr of off-diagonal terms. at1;at2;Dij;RvdW;alfa;ro(sigma);r
  1  2   0.1253   1.5717   9.9736   1.2057  -1.0000  -1.0000
  2  3   0.1125   1.6311   8.7528   1.0929  -1.0000  -1.0000
  1  3   0.0953   1.7397   8.8986   1.4256   1.1067   1.1265
  1  4   0.1926   1.9148  10.0550   1.3376   1.1027   0.9621
  2  4   0.1017   1.7755   9.6088   1.3696  -1.0000  -1.0000
  1  5   0.1495   2.0794  12.2376   0.0100   1.4060  -1.0000
  2  5   0.1361   1.5875  11.9875   1.4900  -1.0000  -1.0000
  3  5   0.2011   2.0377  10.4646   1.6025   1.4785   1.6595
  4  5   0.2161   1.8729   9.9069   2.0896   1.6848  -1.0000
  3  4   0.1869   2.0146  11.0000   1.5197   1.3888  -1.0000
  1  6   0.0800   1.7085  10.0895   1.5504   1.4005  -1.0000
  2  6   0.0366   1.7306  11.1019   1.2270  -1.0000  -1.0000
  3  6   0.0500   1.8000  11.6139   1.4652  -1.0000  -1.0000
  4  6   0.1664   1.7078  11.8610   1.7692  -1.0000  -1.0000
  5  6   0.3188   2.0391  11.1208   2.3703  -1.0000  -1.0000
  1  7   0.0270   2.4124  11.4640   1.7840   1.0000   1.0000
  2  7   0.1149   1.4658  11.0886   1.3337  -1.0000  -1.0000
  3  7   0.0401   1.6839  11.4342   1.8313   1.0000   1.0000
  4  7   0.2114   2.0191  10.2498   2.0765  -1.0000  -1.0000
  1  8   0.0956   1.7010  11.7436   1.3003   1.1889  -1.0000
  2  8   0.0472   1.4236  11.8887   1.1333  -1.0000  -1.0000
  3  8   0.0907   2.3192   9.8579   1.3103   1.2629  -1.0000
  4  8   0.1000   2.0000  10.0000  -1.0000  -1.0000  -1.0000
  1  9   0.1071   1.6243  11.0402   1.3176  -1.0000  -1.0000
  2  9   0.0431   1.6504  10.3632   1.0000  -1.0000  -1.0000
  7  9   0.1000   1.7500  10.5000   0.0001  -1.0000  -1.0000
  8  9   0.0830   1.7419  10.8641   1.3592  -1.0000  -1.0000
  9 10   0.1211   1.7575   9.6653   1.3555  -1.0000  -1.0000
127    ! Nr of angles. at1;at2;at3;Thetao,o;p(val1);p(val2);p(coa1);
  1  1  1  76.1370  34.6920   1.1328   0.0000   0.0050   0.3556   1.8065
  1  1  2  68.0572   9.9461   4.7000   0.0000   0.4566   0.0000   1.8532
  2  1  2  65.6815  35.0000   1.8622   0.0000   0.0490   0.0000   1.0937
  1  2  2   0.0000   4.0000   7.2043   0.0000   0.0000   0.0000   1.0728
  1  2  1   0.0000   3.4110   7.7350   0.0000   0.0000   0.0000   1.0400
  2  2  2   0.0000  30.0000   5.6235   0.0000   0.0000   0.0000   1.0400
  1  1  3  78.3624  13.0773   9.0480   0.0000   0.1270  52.1129   2.3964
  3  1  3  76.7101  24.3833   5.8613 -21.8559   2.6395 -32.6534   3.6179
  2  1  3  79.1288  30.0000   1.4632   0.0000   0.2065   0.0000   2.0000
  1  3  1  80.7352  16.4130   4.9987   0.0000   0.0843   0.0000   1.0137
  1  3  3  85.4436  14.4937   3.9928   0.0000   1.4350  44.5320   1.1348
  3  3  3  89.9282  32.1199   2.7181   0.0000   0.3323  57.6122   1.0000
  1  3  2  82.9640  32.4874   0.8777   0.0000   0.9627   0.0000   1.0010
  2  3  3  85.7838  17.3139   1.9157   0.0000   3.6306   0.0000   2.1858
  2  3  2  84.2527  33.1226   0.6730   0.0000   0.7238   0.0000   2.4348
  1  2  3   0.0000  14.4588   3.1507   0.0000   3.4571   0.0000   1.0149
  3  2  3   0.0000   0.9696   3.6303   0.0000   0.0000   0.0000   1.6987
  2  2  3   0.0000   0.5797   1.9739   0.0000   0.0000   0.0000   2.4494
  3  5  3  80.0647  49.0226   1.1861   0.7271   0.1000   0.0000   1.5321
  1  2  4   0.0000   0.0019   6.0000   0.0000   0.0000   0.0000   1.0400
  1  1  4  66.9777  36.6787   3.9471   0.1463   0.7266   0.0000   1.3415
  1  4  1  65.0000  11.2500   6.1045   0.1463   1.7644   0.0000   1.8642
  2  1  4  30.9196  11.3010   0.5535   0.0000   0.0050   0.0000   1.9267
  1  4  2 100.0000  14.2598   4.2424   0.0000   0.0050   0.0000   3.0000
  1  4  4  92.3921   5.2669   6.7198   0.1463   0.0050   0.0000   2.9982
  2  4  2  92.1229  42.8350   0.6163   0.0000   1.0235   0.0000   1.0010
  2  4  4  70.9476   9.9024   0.6923   0.0000   0.2031   0.0000   2.9811
  5  3  5  16.5418  38.3796   0.5347   0.0000   0.1000   0.0000   2.3535
  3  3  5  34.0844  11.5602   1.5428   0.0000   0.4319   0.0000   1.0500
  3  5  5   6.0985   0.0302   0.1000   0.0000   0.6142   0.0000   1.7575
  2  3  5  88.3222   7.1767   2.4747   0.0000   0.6219   0.0000   3.1507
  1  3  5  76.5850   8.7797   0.8099   0.0000   2.5889   0.0000   1.0500
  4  5  4  66.1778  17.0744   4.2862   0.0984   1.4056   0.0000   1.7545
  5  4  5  35.4696  10.5159   5.6990   0.0000   3.9985   0.0000   1.3642
```

4	4	5	90.0000	32.0246	1.1683	0.0000	3.9500	0.0000	1.3617
4	5	5	41.9144	0.5409	7.1700	0.0000	3.4295	0.0000	3.2326
2	4	5	90.0000	20.3126	0.7222	0.0000	0.6873	0.0000	2.2146
4	4	4	70.3671	5.7180	7.0000	0.0000	0.3683	0.0000	2.4869
2	5	5	57.6230	6.3083	5.0722	0.0000	0.6873	0.0000	1.5510
2	5	4	54.6337	8.6317	6.9912	0.0000	1.6873	0.0000	2.8674
2	5	2	76.2482	11.2841	7.6230	0.0000	0.9375	0.0000	1.0586
3	4	3	73.1328	40.1854	1.2970	-1.0365	0.0101	0.0000	1.0010
1	4	3	78.7291	29.2617	6.9375	0.0000	0.0197	0.0000	1.2505
1	3	4	83.3532	22.9357	0.8136	0.0000	1.1543	0.0000	2.6844
3	3	4	70.2283	45.0000	6.1591	0.0000	2.7147	0.0000	1.0010
2	3	4	45.6742	13.4413	1.5725	0.0000	0.7737	0.0000	2.6616
4	2	4	0.0000	7.5000	2.0000	0.0000	0.0000	0.0000	1.0400
4	2	5	0.0000	7.5000	2.0000	0.0000	0.0000	0.0000	1.0400
5	2	5	0.0000	7.5000	2.0000	0.0000	0.0000	0.0000	1.0400
1	6	1	62.5000	16.6806	0.7981	0.0000	0.9630	0.0000	1.0711
1	1	6	87.6241	12.6504	1.8145	0.0000	0.6154	0.0000	1.5298
6	1	6	100.0000	40.4895	1.6455	0.0000	0.0100	0.0000	1.7667
1	6	6	5.0994	3.1824	0.7016	0.0000	0.7465	0.0000	2.2665
3	6	3	28.9047	27.3847	2.5790	0.0000	0.1078	0.0000	2.4145
3	3	6	90.0000	39.1857	4.8200	0.0000	0.9067	0.0000	1.9533
6	3	6	51.5671	2.9451	0.6657	0.0000	1.6341	0.0000	1.9057
3	6	6	56.7026	3.2665	4.3063	0.0000	0.6729	0.0000	2.7490
2	6	2	106.3969	30.0000	0.9614	0.0000	1.9664	0.0000	2.2693
2	2	6	0.0000	26.3327	4.6867	0.0000	0.8177	0.0000	1.0404
6	2	6	0.0000	60.0000	1.8471	0.0000	0.6331	0.0000	1.8931
2	6	6	30.3748	1.0000	4.8528	0.0000	0.1019	0.0000	3.1660
2	6	6	180.0000	-27.2489	8.3752	0.0000	0.8112	0.0000	1.0004
1	6	2	97.5742	10.9373	2.5200	0.0000	1.8558	0.0000	1.0000
1	2	6	0.0000	0.2811	1.1741	0.0000	0.9136	0.0000	3.8138
2	1	6	84.0006	45.0000	0.6271	0.0000	3.0000	0.0000	1.0000
2	3	6	28.4774	12.0885	3.2396	0.5000	0.0778	0.0000	1.6733
1	6	3	70.0000	25.0000	1.0000	0.0000	1.0000	0.0000	1.2500
1	3	6	70.0000	25.0000	1.0000	0.0000	1.0000	0.0000	1.2500
3	1	6	70.0000	25.0000	1.0000	0.0000	1.0000	0.0000	1.2500
3	2	6	0.0000	1.0000	1.3402	0.5000	0.0500	0.0000	1.5379
3	5	4	75.0000	25.0000	2.0000	0.0984	1.0000	0.0000	1.5000
4	3	5	35.0000	12.5000	1.5000	0.0000	0.5000	0.0000	1.0500
3	4	5	90.0000	30.0000	1.2500	0.0000	3.0000	0.0000	1.3000
3	4	4	70.0000	45.0000	3.0000	0.0000	2.0000	0.0000	1.1000
2	5	3	70.0000	12.0000	4.0000	0.0000	1.0000	0.0000	1.2500
3	2	5	0.0000	15.0000	2.0000	0.0000	0.0000	0.0000	1.0500
4	6	4	2.7962	7.1073	0.5589	0.0000	0.0554	0.0000	1.1473
6	4	6	92.9945	26.8345	0.9189	0.0000	0.0100	0.0000	1.4683
4	6	6	48.7356	9.9227	0.1206	0.0000	0.0893	0.0000	1.1108
4	4	6	64.5223	7.2562	5.2298	0.0000	0.5459	0.0000	1.0400
2	4	6	83.4937	16.7605	0.8242	0.5000	0.5409	0.0000	1.1378
4	2	6	0.0000	10.0000	1.0000	0.5000	0.2500	0.0000	1.5000
5	4	6	61.8263	20.8696	0.2450	0.0000	0.7429	0.0000	1.0400
4	5	6	60.0000	1.0000	1.0000	0.0000	1.0000	0.0000	1.2500
4	6	5	60.0000	1.0000	1.0000	0.0000	1.0000	0.0000	1.2500
5	3	6	44.9106	2.7940	0.5834	0.0000	0.9597	0.0000	1.3151
3	5	6	60.0000	1.0000	1.0000	0.0000	1.0000	0.0000	1.2500
3	6	5	60.0000	1.0000	1.0000	0.0000	1.0000	0.0000	1.2500
2	7	2	25.9881	0.0100	1.8827	0.0000	0.6581	0.0000	1.1500
7	2	7	0.0000	3.6249	1.0000	0.0000	1.0000	0.0000	1.2500
4	7	4	66.4795	8.0479	5.0000	0.0000	1.3950	0.0000	1.6004
7	4	7	75.6682	7.4119	3.8037	0.0000	0.0100	0.0000	3.4502
4	4	7	100.0000	5.2297	0.8528	0.0000	1.0382	0.0000	1.1870
4	7	7	78.8126	1.9499	3.4361	0.0000	0.0100	0.0000	3.6457
2	4	7	70.0000	10.0000	1.0000	0.0000	1.0000	0.0000	1.2500
3	7	3	10.0000	0.0000	1.0000	0.0000	1.0000	0.0000	1.0000
3	3	7	79.2012	30.0000	2.0000	0.0000	1.0000	0.0000	1.0000
1	3	7	90.0000	14.7654	1.7889	0.0000	1.7537	0.0000	1.0000
3	1	7	88.4731	0.4260	0.7999	0.0000	0.9856	0.0000	1.9800
7	3	7	40.0000	1.0000	1.0000	0.0000	1.0000	0.0000	1.0000
2	8	2	50.0000	26.9005	1.7315	0.0000	0.1848	0.0000	1.0400
2	8	8	55.2500	36.5272	6.0000	0.0000	0.4281	0.0000	2.1149
2	2	8	0.0000	10.4651	0.1000	0.0000	0.0000	0.0000	3.0000
2	3	8	75.9746	10.9523	0.8687	0.0000	1.8256	0.0000	2.9875
2	8	3	65.0000	40.0000	6.0000	0.0000	0.1000	0.0000	3.0000
3	8	3	50.4947	12.1095	3.5926	0.0000	3.0000	35.0000	1.0400
8	3	8	90.0000	40.0000	4.7885	0.0000	2.7146	0.0000	1.0400
3	2	8	52.0162	2.5267	0.3146	0.0000	2.2070	0.0000	2.9111

```
  3  3  8  90.0000  27.7492   6.0000   0.0000   0.1870   0.0000   1.0400
  8  2  8   0.5000   3.4405   0.9580   0.0000   0.8031   0.0000   1.0000
  8  8  8  60.9386  12.9033   7.8607   0.0000   1.7515   0.0000   2.2405
  3  8  8  70.7224   5.3644   3.4424   0.0000   0.8219   0.0000   2.8000
  1  1  8  30.0491  23.9749   3.2341   0.0000   1.0000   0.0000   1.0000
  1  8  1  80.6555  40.0000   5.6273   0.0000   1.0000   0.0000   3.7089
  1  8  8  70.5217  39.3118   7.9958   0.0000   1.0000   0.0000   1.0000
  8  1  8  47.0626   4.5590   5.6859   0.0000   1.0000   0.0000   1.4685
  1  8  3  75.0000  30.0000   2.0000   0.0000   1.0000   0.0000   2.0000
  1  8  2  65.0000  35.0000   4.0000   0.0000   0.5000   0.0000   2.0000
  1  1  9  72.8393  34.6130   5.4732   0.0000   1.6548   0.0000   3.4929
  9  1  9  79.9557  40.0000   5.2564   0.0000   2.8000   0.0000   1.0000
  1  9  1   0.0000  19.9962   3.2299   0.0000   2.1012   0.0000   1.1537
  1  9  9   0.0000  25.0000   1.0000   0.0000   1.0000   0.0000   1.0400
  2  1  9  75.2593  17.6832   2.7253   0.0000   1.8603   0.0000   1.0000
  9  8  9  65.0386  28.8263   2.2480   0.0000   1.1021   0.0000   1.0400
  8  9  9  70.0000  28.7353   1.2918   0.0000   1.0913   0.0000   1.0400
  8  8  9  70.0000  25.0000   2.5000   0.0000   1.0000   0.0000   1.0400
  9 10  9  92.7358  25.0000   2.5000   0.0000   1.1355   0.0000   1.1514
 62    ! Nr of torsions. at1;at2;at3;at4;;V1;V2;V3;p(tor1);p(cot1);n
  1  1  1  1   2.0474  32.6719   0.5282  -9.0000  -2.6449   0.0000   0.0000
  1  1  1  2   1.6328  78.4995  -0.1514  -6.9161  -2.9986   0.0000   0.0000
  2  1  1  2   2.4142  78.7025   0.3506  -8.8640  -6.9283   0.0000   0.0000
  1  1  1  3  -0.7104  22.6038   0.5309  -2.0000  -0.6614   0.0000   0.0000
  2  1  1  3   1.9323  52.9368   0.6554  -8.8118  -3.9854   0.0000   0.0000
  3  1  1  3  -1.2500   1.1248  -0.1230  -9.9453  -3.9000   0.0000   0.0000
  1  1  3  1  -0.6848  56.7751  -1.2733  -2.2937  -4.0000   0.0000   0.0000
  1  1  3  2  -1.4557  78.6279   0.9945  -3.2742  -2.4240   0.0000   0.0000
  2  1  3  1   0.6928  78.1546   0.5608  -3.1713  -3.7301   0.0000   0.0000
  2  1  3  2  -1.4343  77.0699   0.9875  -3.4139  -1.4053   0.0000   0.0000
  1  1  3  3   0.5153   2.1584   0.2000  -6.5859  -8.0000   0.0000   0.0000
  2  1  3  3   0.2018  80.0000   0.3778  -2.5000  -7.8750   0.0000   0.0000
  3  1  3  1  -3.9875  79.2591   1.0000  -2.4206  -3.9342   0.0000   0.0000
  3  1  3  2  -1.1000  78.8002  -1.0000  -2.6282  -4.0000   0.0000   0.0000
  3  1  3  3  -1.0000  83.5323   4.3660  -2.6805  -1.2938   0.0000   0.0000
  1  3  3  1   3.4682   0.0781   0.9887  -6.1195  -0.5004   0.0000   0.0000
  1  3  3  2   1.0000  16.5478  -1.0313  -2.0000  -2.6888   0.0000   0.0000
  2  3  3  2   4.0818  -3.2744  -0.9664  -7.1634  -3.0000   0.0000   0.0000
  1  3  3  3   4.2014 -10.0642   1.8690  -2.4805  -2.5000   0.0000   0.0000
  2  3  3  3   1.0000 -10.0500  -1.0000  -2.1946  -0.5300   0.0000   0.0000
  3  3  3  3   1.0000   1.6871   3.0000  -6.2660  -0.5500   0.0000   0.0000
  0  1  2  0   0.0000   0.0000   0.0000   0.0000   0.0000   0.0000   0.0000
  0  2  2  0   0.0000   0.0000   0.0000   0.0000   0.0000   0.0000   0.0000
  0  2  3  0   0.0000   0.1000   0.0200  -2.5415   0.0000   0.0000   0.0000
  0  1  1  0   0.0000  50.0000   0.3000  -4.0000  -2.0000   0.0000   0.0000
  0  3  3  0   0.5511  25.4150   1.1330  -5.1903  -1.0000   0.0000   0.0000
  0  1  4  0  -0.2500  80.0000   1.0000  -3.0140  -2.4381   0.0000   0.0000
  0  2  4  0   0.0000   0.0000   0.0000   0.0000   0.0000   0.0000   0.0000
  4  4  4  4   1.8235 -11.0688  -0.4137  -2.7875  -1.3961   0.0000   0.0000
  2  1  3  5   2.1344  29.9850   0.3398  -3.1459  -2.1000   0.0000   0.0000
  1  1  3  5   0.4573  10.0000   1.0000  -7.3632  -2.1000   0.0000   0.0000
  2  3  5  3   0.3709  10.0000   0.9625  -9.0000  -1.0000   0.0000   0.0000
  2  3  4  3   2.5000   2.5000   0.2237 -10.0000   0.0000   0.0000   0.0000
  0  3  4  0   0.5000  50.0000   0.5000 -10.0000   0.0000   0.0000   0.0000
  3  4  4  4   0.2500  90.0000   0.5000  -6.0000   0.0000   0.0000   0.0000
  3  4  4  3   0.2500  90.0000   0.5000  -6.0000   0.0000   0.0000   0.0000
  1  4  4  1   0.0000  50.0000   0.0000  -8.0000   0.0000   0.0000   0.0000
  1  4  4  2   0.0000  50.0000   0.0000  -8.0000   0.0000   0.0000   0.0000
  2  4  4  2   0.0000  50.0000   0.0000  -8.0000   0.0000   0.0000   0.0000
  1  1  1  6   0.0000   5.0000   0.4000  -6.0000   0.0000   0.0000   0.0000
  6  1  1  6   0.0000  44.3024   0.4000  -4.0000   0.0000   0.0000   0.0000
  2  1  1  6   0.0000  21.7038   0.0100  -4.0000   0.0000   0.0000   0.0000
  2  1  6  1   0.0000   5.2500   0.0100  -6.0000   0.0000   0.0000   0.0000
  1  1  6  1   0.0000   5.1676   0.0100  -5.9539   0.0000   0.0000   0.0000
  1  1  6  2   0.0000   5.1676   0.0100  -5.9539   0.0000   0.0000   0.0000
  6  3  3  6   0.0509  30.0000   0.5000  -4.0000   0.0000   0.0000   0.0000
  4  4  4  7   0.0000   2.0000   0.0100  -9.0000   0.0000   0.0000   0.0000
  7  4  4  7   0.0000  20.0000   0.0100  -5.0000   0.0000   0.0000   0.0000
  0  8  8  0   0.0000  42.3911  -0.3192  -4.3105   0.0000   0.0000   0.0000
  0  3  8  0  -2.0000  48.7726  -0.5000  -2.5000   0.0000   0.0000   0.0000
  8  3  3  8   2.0000  75.0000   0.3000  -5.0000   0.0000   0.0000   0.0000
  0  1  8  0   0.0000  30.0000  -0.1000  -5.0000   0.0000   0.0000   0.0000
  1  1  1  8   0.0000   2.0000   0.0000  -6.0000   0.0000   0.0000   0.0000
  8  1  1  8   0.0000   2.0000   0.0000  -6.0000   0.0000   0.0000   0.0000
```

```
1  1  1  9   0.0000  52.8812  -0.2000  -5.7302  -2.0000   0.0000   0.0000
2  1  1  9   0.0000  88.0596  -0.0534  -7.0044  -2.0000   0.0000   0.0000
9  1  1  9   0.0000  90.0000   0.3121  -6.4061  -2.0000   0.0000   0.0000
0  1  9  0   0.0000  50.0000   0.5000  -6.0000   0.0000   0.0000   0.0000
0  9  9  0   0.0000   1.0000   0.1000  -6.0000   0.0000   0.0000   0.0000
1  1  3  7   0.0000  50.0000   0.2000  -4.0000   0.0000   0.0000   0.0000
2  1  3  7   0.0000  50.0000   0.2000  -4.0000   0.0000   0.0000   0.0000
3  1  3  7   0.0000  50.0000   0.2000  -4.0000   0.0000   0.0000   0.0000
4    ! Nr of hydrogen bonds. at1;at2;at3;r(hb);p(hb1);p(hb2);p(hb3
3  2  3   1.8130  -3.5409   2.3815  21.9463
3  2  4   2.0000  -2.0000   2.0000  15.0000
4  2  3   2.0000  -2.0000   2.0000  15.0000
4  2  4   2.0000  -2.0000   2.0000  15.0000
```
